# Artificial Intelligence in Eating Disorder Treatment: A Qualitative Analysis of Clinical Opportunities, Barriers, and Ethical Considerations From Multi‐Disciplinary Focus Groups

**DOI:** 10.1002/eat.24579

**Published:** 2025-10-20

**Authors:** J. Maas, S. Franssen, M. Petkovic, S. Cardona Cano, A. E. Dingemans, A. M. van Oosterzee, M. C. T. Slof‐Op ’t Landt, E. Talavera Martinez, C. M. J. M. Vreeswijk, M. Simeunovic‐Ostojic

**Affiliations:** ^1^ Center for Eating Disorders Helmond, Mental Health Center Region Oost‐Brabant Helmond the Netherlands; ^2^ Department of Medical and Clinical Psychology Tilburg University Tilburg the Netherlands; ^3^ Department of M & CS Technical University of Eindhoven Eindhoven the Netherlands; ^4^ Philips Hospital Patient Monitoring Eindhoven the Netherlands; ^5^ Youz Rotterdam the Netherlands; ^6^ GGZ Rivierduinen Eating Disorders Ursula Leiden the Netherlands; ^7^ Faculty of Humanities, Utrecht University Utrecht the Netherlands; ^8^ Data Management and Biometrics, University of Twente Enschede the Netherlands

**Keywords:** artificial intelligence (AI), clinical challenges, clinical opportunities, eating disorders, ethical challenges, focus groups, qualitative analysis, treatment

## Abstract

**Objective:**

This study explored eating disorder and Artificial Intelligence (AI) professionals' perspectives on how AI might support eating disorder treatment. Successful implementation requires insight into implementation partners' perspectives.

**Method:**

This study is an explorative qualitative analysis of two interdisciplinary focus groups (consisting of 22 eating disorder and AI professionals in total). Qualitative analysis with ATLAS.ti using a hybrid thematic analysis approach combined deductive coding with inductive theme development. The groups discussed (1) the opportunities and challenges—including ethical and safety considerations—of AI in eating disorder care, and (2) the types of evidence and evaluation frameworks required for adoption in practice.

**Results:**

Themes were categorized into “opportunities,” “challenges,” “concerns,” “solutions,” and “evidence needed.” Opportunities focused on AI's potential to enhance efficiency, support treatment delivery and monitoring, and reduce human error. Challenges concerned barriers to adoption in clinical practice, responsibility, and explainability. Concerns included ethical and legal risks, also related to data sharing. Proposed solutions emphasized the need for human oversight, cross‐sector collaboration, and clinician training. With regard to evidence needed, participants mentioned safety and accuracy, and the need for scientific testing and validation.

**Discussion:**

This study highlighted the potential and complexity of integrating AI into eating disorder care from the viewpoint of eating disorder and AI professionals. While there is value in AI in improving efficiency and clinical support, successful implementation requires addressing ethical concerns, legal uncertainty, and infrastructural barriers. Collaboration across disciplines, rigorous validation, and clinician involvement are essential to ensure that AI applications are safe, meaningful, and ethically sound.

**Summary**

- By bringing together experts in artificial intelligence (AI) and eating disorder care, we identified both opportunities and ethical concerns on how AI may support the treatment of eating disorders.
- The findings highlight the importance of careful implementation, clinician involvement, and human oversight to ensure that AI tools are safe, helpful, and aligned with the needs of patients and professionals.

## 1 Introduction

Eating disorders, such as anorexia nervosa, bulimia nervosa, and binge eating disorder, are associated with an often chronic and severe course of disease, over half of the patients not achieving recovery, and high mortality rates (Solmi et al. 2024). Most individuals suffer from co‐occurring psychiatric disorders, while medical comorbidities and complications are also frequently present (Hambleton et al. 2022), indicating the complexity of these conditions. The complexity is furthermore illustrated by the fact that eating disorders have a multifactorial onset, meaning that these conditions emerge from the dynamic interaction between multiple factors (e.g., genetic vulnerability, biological mechanisms, psychological traits, and environmental and social contexts, including adverse or traumatic life events), and the pathways leading to illness are likely highly individualized (Keski‐Rahkonen 2024). As neurobiological and psychosocial maintaining factors develop as the disease progresses, the chances of recovery decrease over time (Ambwani et al. 2020). Early diagnosis and treatment are therefore of utmost importance.

Artificial intelligence (AI) holds significant promise for enhancing the early detection and treatment of eating disorders by leveraging data‐driven screening tools and predictive models to support more personalized care (Linardon et al. 2025). In the context of early detection, machine learning techniques can help identify individuals who are at heightened risk for somatic complications or who are most likely to benefit from specific treatment approaches (Fardouly et al. 2022; Ghosh et al. 2024). Beyond this, AI offers further opportunities for early detection, such as processing and integrating data from wearable devices into clinical care and decision‐making. AI may also improve treatment delivery by supporting clinicians in administrative tasks such as writing a summary of their session or intake interview through text technology, using large language models, and by enabling the use of chatbots to facilitate initial engagement or early intervention (Sharp et al. 2025). By automating certain clinical and administrative tasks, these technologies may help alleviate some of the pressure caused by workforce shortages. To summarize, these opportunities are both promising and urgently needed, given the high demand for specialized treatment and the limited availability of qualified care. Long waiting lists remain a persistent issue, exacerbated not only by workforce shortages but also by the administrative burden placed on clinicians (Linardon et al. 2025). Reflecting this need, many clinicians have already started adopting AI in their daily practice. Linardon and colleagues, for example, reported that 59% of the clinicians they surveyed had used AI to enhance professional efficiency and support, with ChatGPT being the most frequently mentioned tool.

Nonetheless, the application of AI in the treatment of eating disorders is not without risk. A frequently cited example is the case of the therapy chatbot Tessa. Initially, Tessa was developed as a rule‐based chatbot and tested in a randomized controlled trial, where it showed promise in providing structured, evidence‐based support for eating disorder prevention (Fitzsimmons‐Craft et al. 2022). However, when the chatbot was later implemented on NEDA's website by a mental health chatbot company, a generative AI feature was erroneously enabled. This version began providing dieting advice and even encouraging weight loss, ultimately leading to its suspension (Jargon 2023). The incident illustrates not that a rigorously tested rule‐based intervention failed, but rather the risks of deploying general‐purpose generative AI systems in highly sensitive contexts without safeguards or clinical oversight. The risks therefore lie not only in the technology itself but also in the way systems are implemented, monitored, and communicated to users. These cases show the urgency of distinguishing between different types of AI systems, clarifying their intended scope of use, and ensuring robust mechanisms to mitigate potential harms. Rather than debating whether to adopt AI in mental health care, the pressing task is to determine how to integrate it responsibly, aligning its use with ethical standards and safeguarding the best interests of patients, clinicians, and other partners.

To address questions raised by the rapid development of AI applications and the introduction of these in clinical care in a grounded and interdisciplinary way, we organized an expert meeting that brought together professionals working with AI and eating disorder experts (both clinicians and researchers). The aim of this meeting was to explore opportunities, challenges, and ethical considerations surrounding AI implementation in this sensitive field. Open and ongoing dialogue between clinicians and AI researchers is vital for designing future plans for the adoption of AI solutions that align with clinical needs. This is especially important given the risk that the two fields may not speak the same language, a disconnect that can hinder effective collaboration. Participants of the expert meeting explored multidisciplinary perspectives on the integration of AI in eating disorder research and treatment in two focus groups, particularly aimed at exploring how AI might support the treatment of eating disorders. Although not formally designed as a qualitative study, the rich discussions yielded valuable insights into the adaptation of AI within clinical practice for eating disorders. Therefore, we report the findings here in the form of an exploratory qualitative analysis.

## 2 Methods

We used the Consolidated criteria for Reporting Qualitative research (COREQ) checklist (Tong et al. 2007) in the reporting of the results. To protect participant confidentiality in accordance with General Data Protection Regulation (GDPR) guidelines (European Union 2016), we anonymized all quotes and reported limited participant characteristics in aggregate form to prevent potential identification, especially given that some participants were also co‐authors.

### 2.1 Participants and Procedure

On September 13, 2024, an expert meeting was held at Eindhoven University of Technology (Eindhoven, the Netherlands), bringing together professionals with expertise in AI and clinicians and researchers specialized in eating disorders. This expert meeting was organized within the framework of an ongoing collaboration between GGZ Oost Brabant (mental healthcare institution in the Netherlands) and Eindhoven University of Technology, as part of the ITEA (Information Technology for European Advancement)–funded project DAISy (Developing AI ecoSystems improving diagnosis and care of mental diseases). The aim of the expert meeting was to exchange knowledge and experiences regarding the current state of AI research and implementation in both domains, and to explore mutual needs, opportunities, and challenges. GGZ Oost Brabant and Eindhoven University of Technology invited experts from their own extended academic, clinical and industry collaborative networks.

In the morning, participants gave short pitches to share about their own ongoing research, implementation experiences, and future visions of AI in their respective fields. The afternoon program consisted of two parallel focus group sessions. Both focus groups lasted approximately 60 min and were held simultaneously in separate rooms. Each focus group was deliberately composed to ensure a balanced distribution of clinical professionals (e.g., psychologists, psychiatrists, nurses, clinical researchers) and AI professionals (e.g., software developers, AI researchers, computer scientists). Group A consisted of 12 participants (eight clinical professionals and four AI professionals, aged 25–66), and Group B consisted of 10 participants (eight clinical professionals and two AI professionals, aged 34–53). Although the focus groups were initially designed as exploratory brainstorming sessions to strengthen the interdisciplinary network and identify opportunities for future collaboration and joint funding applications, the focus group discussions were also audio‐recorded and later analyzed for the purpose of this study. Prior to the recordings, all participants provided verbal informed consent for the sessions to be recorded and potentially used for research purposes. After the sessions, we obtained written informed consent from all individuals. No participants declined participation after being informed about the research purpose and procedures. To further ensure ethical compliance, we received approval from the Committee for Scientific Research (Commissie Wetenschappelijk Onderzoek) of GGZ Oost Brabant. This committee confirmed that the study met the criteria for responsible research practice within our institutional and national guidelines.

Each focus group was moderated by one of two researchers: either a female psychiatry resident and researcher in the field of eating disorders, or a female post‐doctoral researcher in mathematics and computer science. Although they were not formally trained as facilitators, both had (research) experience relevant to the topic. They guided the discussion and encouraged participation. The moderators were not involved in the data analysis, and any personal input they may have provided during the sessions was not included in the coding process. The moderators guided the discussion to cover both central topics. These topics, printed on a large sheet visible throughout the session, were: (1) the challenges and opportunities—including ethical and safety considerations—of integrating AI into routine clinical practice, and (2) the types of evidence clinicians require to confidently adopt AI‐based tools, along with appropriate methods for evaluating such tools. These prompts served as open discussion guidelines; no formal interview guide was used.

## 3 Data Analysis

Audio recordings of the focus groups were transcribed verbatim and anonymized. To mitigate bias and increase reflexive distance, the analysis was conducted by two researchers (J.M., female psychologist and senior researcher at the Centre for Eating Disorders; and S.F., female neuroscientist and senior researcher at the Centre for Eating Disorders): one of them had not attended either focus group and was thus not involved in the discussions. This ensured that at least one analyst maintained an outsider perspective throughout the analysis. Thematic analysis was conducted following the six‐phase approach described by Braun and Clarke (2006), using the qualitative data analysis software ATLAS.ti. These phases include familiarization with the data, generation of codes (labels capturing relevant features of the data), combining codes into themes (broader patterns of meaning), reviewing themes, determining the significance of themes, and reporting findings. Transcripts were not returned to participants for questions or comments.

While the central topics discussed in the focus groups served as the initial framework for the analysis, the researchers adopted a hybrid approach (Fereday and Muir‐Cochrane 2006), combining both deductive and inductive thematic analysis. This meant that, although coding was initially guided by the predefined focus areas, additional codes and themes were developed inductively based on patterns emerging directly from the data. This approach allowed for a deeper and more nuanced understanding of participants' perspectives beyond the original discussion prompts. After initial coding, the researchers met to compare codes, reflect on patterns, and collaboratively develop a thematic structure. This included discussions about the grouping of codes into themes and subthemes, decisions about whether certain codes overlapped or warranted separation, and appropriate naming of the themes. One researcher then produced a thematic map, which was reviewed and refined by the second coder. All decisions were grounded in the original focus group questions and discussed transparently. Discrepancies were resolved through discussion and consensus. Throughout this process, coding was treated as an interpretative and iterative process, not as a mechanical act of categorization. Consistent with the reflexive approach, the focus was not on inter‐rater reliability but on depth, coherence, and conceptual clarity of the themes. Although the analysis was primarily qualitative, code frequencies were calculated to provide insight into how often certain topics were raised in each focus group.

## 4 Results

The initial thematic framework was informed by the discussion guide and consisted of five overarching themes: (1) opportunities for integrating AI in clinical practice, (2) challenges, (3) ethical and safety considerations, (4) types of evidence needed for clinical adoption, and (5) appropriate methods for evaluation. As analysis progressed, it became clear that these overarching themes did not fully capture the complexity of the data. In particular, many of the opportunities and challenges discussed by participants mapped onto more specific subthemes, prompting us to introduce an additional hierarchical layer in the coding structure. Several codes were grouped under clearly defined subthemes (such as “use of AI in treatment” under “opportunities,” and “adoption in practice” and “human‐AI interaction in clinical decision‐making” under “challenges”), while others remained standalone due to their conceptual distinctiveness. Moreover, within the original conceptualized overarching theme of “challenges,” we identified an important distinction between barriers (i.e., practical obstacles that could potentially be addressed) and concerns (i.e., fundamental worries or unresolved issues, often with ethical implications). We therefore created two separate overarching themes for “challenges” and “concerns.” Ethical and safety considerations, initially coded as a separate theme, were furthermore reclassified under “concerns.” Participants also frequently proposed solutions in response to the challenges discussed. For example, they mentioned “human in the loop” as a solution when discussing the challenges related to “responsibility and accountability” (see below for more detail). These were not incorporated under “opportunities,” as they often directly responded to specific challenges or concerns. Instead, they were coded separately as “solutions.” Finally, the overarching themes of “evidence needed” and “evaluation” were combined, as participants often discussed these aspects in an integrated way. The themes and subthemes, as well as codes and their frequencies, are presented in Table 1. Table 2 reports representative examples of quotes. To protect participant confidentiality, quotes are labeled only with the focus group letter and the participant's background (e.g., A‐AI for a participant from the AI/technical domain in Group A; B‐C for a participant from the clinical domain in Group B).

**TABLE 1:** Overarching themes, subthemes, and codes, including frequencies.

Overarching theme	Subtheme	Code	*N* mentioned (Group A)	*N* mentioned (Group B)
Opportunities	Use of AI in treatment	Efficiency/administration	5	2
	Use of AI in treatment	Intervention	5	0
	Use of AI in treatment	Predictors	5	1
	Use of AI in treatment	Monitoring	0	3
		AI more accurate/objective than human	4	2
		Help more patients	1	0
		Social media problems create urgency	0	1
Challenges	Adoption in practice	Competing with existing platforms	4	3
	Adoption in practice	Standardization & generalizability	5	1
	Adoption in practice	Funding	0	4
	Adoption in practice	Implementation	1	3
	Adoption in practice	Usability	1	0
	Human–AI interaction in clinical decision‐making	Responsibility & accountability	1	4
	Human–AI interaction in clinical decision‐making	Explainability	0	4
	Human–AI interaction in clinical decision‐making	Too much trust	3	0
Concerns		Ethical & safety considerations	6	6
		Data sharing	1	4
		Legal/liability	0	6
Solutions		Human in the loop	6	5
		Collaboration	2	6
		Train clinicians	3	1
Evidence needed/evaluation		Safe & accurate	12	1
		Testing & validation	5	7

**TABLE 2:** Representative examples of quotes.

(Overarching theme>Subtheme>)Code	Representative quotes
(Opportunities>Use of AI in treatment>)Efficiency/administration	“In the end reading something and checking will always be quicker than writing everything yourself. I don't want to just think of challenges, but I think there really are also opportunities to make our care more efficient.” (A–C) “We need for example a tool that can do your intake. It will be wonderful, because it takes a lot of time.” (B–C)
(Opportunities>Use of AI in treatment>)Intervention	“It will be helpful if the patient gets a notification when the levels of stress are rising and you can prevent it. For example, one of the important factors in CBT is writing it down, so you're just decreasing momentum and with exercises, for example, like an AI‐tool on the phone, you can prolong the moment to the binge for example, and that could help them in the treatment.” (A–C)
(Opportunities>Use of AI in treatment>)Predictors	“I once heard on TV, someone who said that he was very depressed over the past six years and he put all his diary entries into ChatGPT. He asked the question “What are predictors of my depressive episodes?” And ChatGPT apparently gave quite a good overview over this bulk of data over the past six years.” (A–C) “Like if you treat patients for, I don't know, 10–12 weeks and then you don't see any progress in treatment. Then should you stop or not, should you do something different… It will be something very interesting to look at. Because I think that we, as therapists, tend to go on too long and maybe we should change earlier, based on information. That the system knows it won't get better, for example, so…” (B–C)
(Opportunities>Use of AI in treatment>)Monitoring	“In the clinic for example, with the level of sugar in the blood. It will be nice to monitor those aspects.” (B–C)
(Opportunities>)AI more accurate/objective than human	“But is a human decision more accurate and safe than an AI based decision?… if you look at studies, for example with GPs… they're sometimes even more accurate than a physician.” (A–C)
(Opportunities>)Help more patients	“I have a colleague who really says, well, our field is going to change within a few years, so much, and I think can also be very positive, because if you can help a lot of people with this type of intervention and you really have the more severe cases, that you have the time for, for in person contact and the medication…” (A–C)
(Opportunities>)Social media problems create urgency	“I think there are some real ethical opportunities with this particular topic, particularly because it affects women and it seems like something where society should take a lot of responsibility for what's sort of the toxic media environment around body image and stuff. And so I think that can really help justify why you need to do research urgently on this topic, and so I think it's important to always keep that in mind.” (B–AI)
(Challenges>Adoption in practice>)Competing with existing platforms	“For me, the challenges are how are we going to make this more important than TikTok or Instagram? So, how are we going to seduce them to use our AI tools instead of what they have all day long?” (A–C) “I do feel like there's going to be a little bit of an arms race between extra clinical medical AI that's just available out there. As a consumer product or something that people search for themselves on the Internet versus clinically, like official AI products. And so I think that arms race will start to intensify in the next few years because people just get access to stuff on the Internet… And so I think ultimately clinicians need to have something else to offer.” (B–AI)
(Challenges>Adoption in practice>)Standardization & generalizability	“You maybe have a lot of biases in the model because you'll be restricted to maybe you only have a number of eating disorder patients that are 16 years old and then you have one, that's 25. And your model will not be at all fitted to that.” (A–AI) “Also the quality of the data because sometimes we think we have a lot of data, but we go into all of these files and end up finding out that not everyone fills it in the same way.” (A–AI)
(Challenges>Adoption in practice>)Funding	“But I think it comes back to the funding… You have to really do an experimental design and do that with a controlled group of people… And that is where the money was missing to do that part.” (B–AI)
(Challenges>Adoption in practice>)Implementation	“Many of these tests are some things that they were saying would take years for us to just get there and then say then we cannot even start using these tools. While in my field, basically, we first put the tool there, right, and then you see what happens.” (B–AI)
(Challenges>Adoption in practice>)Usability	“If you want to do this, it has to be not a burden for clinicians or patients or caregiver service, it needs to be readily available… Otherwise the threshold to adopt is maybe too high.” (A–C)
(Challenges>Human‐AI interaction in clinical decision‐making>)Responsibility & accountability	“Because it is a part of medical treatment, there has always been a person responsible for it. So at this moment in time you have someone who makes that decision.” (A–C)
(Challenges>Human‐AI interaction in clinical decision‐making>)Explainability	“As soon as you also incorporate brain scan data… within no time you have such a high amount of potential connections… at a stage that it's essentially not explainable anymore to any human what's going on… It makes a lot of intuitive sense that it's ethical for it to be explainable, but if non‐explainable is vastly better, then what happens?” (B‐C)
(Challenges>Human‐AI interaction in clinical decision‐making>)Too much trust	“Speaking about trust, we've spoken a lot about the lack of trust in AI, but within my own generation of young future doctors, I just see like an over‐trust in AI maybe.” (A‐C)
(Concerns>)Ethical & safety considerations	“We have to consider autonomy. So how much autonomy do you want to give to your patients? How much do you want to take away? Sometimes a tool does that in a way that you didn't expect it to do. You might want to empower your patient, so then you take away a little bit of autonomy, but you might give some strength back in another way. Fairness, bias, ableism concerns, I have already heard of a lot of projects that have a normal human as a standard model and then try to model the other humans, which is kind of worrying to me. But also sometimes just impossible not to do. Discrimination, responsibility, privacy, agency, fairness, trustworthiness…” (A–C)
(Concerns>)Data sharing	“I think even at the national level, sharing of data is difficult.” (A–C) “If, for example, she creates a great algorithm for the intake and we use it in our institutions to make it better. Where does that data go?” (B‐AI)
(Concerns>)Legal/liability	“Even if you move just the model around, it may remember some parts and then have already proven that some of the ChatGPTs remember parts of things that are copyrighted. There are big legal battles going on so… It is really complicated.” (B‐AI)
(Solutions>)Human in the loop	“The other thing also that we discussed is really maybe this aspect of assistance, so having this as a sort of, maybe an assistant, that can always be checked by a human who is actually making a decision afterwards.” (A–AI) “If I get all those labs. Then I rely on my intelligence. I use this as a tool. Maybe it will be dangerous if you just choose to use the algorithm. You need the doctor. You need the psychologist.” (B– C)
(Solutions>)Collaboration	“This is the reason I think we need to make this coalition for artificial intelligence for eating disorders because you need to put on paper… a contract, everything, including our intellectual properties and rights and your time and your intellectual properties in the project.” (B, C)
(Solutions>)Train clinicians	“Yeah, so what comes with it maybe also is to train clinicians on which product you develop. Train clinicians, how to use it, how to interpret it, what you can infer from it and what you can't…” (A–C)
(Evidence needed/evaluation>)Safe & accurate	“Yeah, but I think on whichever level it is, what we needed was a lot of more certainty about the validity and experience with it. I think right, like in general, because it's quite new, I think for most of us, especially for clinicians and it's where you can't be 100% safe or sure, but you do need a fair amount of…” (A–C) “As somebody from clinical practice, I think that you really need to be sure that if the patient for example is asking a question about binge eating or vomiting that the automated answer, or the chatbot, or whatever is used, is really giving the correct information. Because I think that it could be hurtful if somebody gets the wrong tips.” (B, C)
(Evidence needed/evaluation>)Testing & validation	“So, at some point it needs to be also data that is readily available, but that you somehow know as a clinician that the people who have developed this have tested their model in several ways and that you know it's reliable and it's accurate” (A–C)

### 4.1 Opportunities

#### 4.1.1 Use of AI in Treatment

Participants in both focus groups identified several potential benefits of integrating AI into treatment. These opportunities largely converged under the broader subtheme “use of AI in treatment,” which included improved efficiency and help with administration, intervention delivery, treatment prediction, and treatment monitoring.

The first key opportunity discussed by both groups was the potential for AI to support treatment delivery through improvements in efficiency and administrative burden (“efficiency/administration”), such as automating parts of clinical documentation or intake procedures. Participants envisioned AI tools that could reduce repetitive tasks, increase objectivity in reporting, and save time. Participants in Group A (but not in Group B) also discussed the potential of AI‐based tools playing a more active role in “intervention” delivery, for instance by supporting patients in moments of high risk. They envisioned AI helping patients regulate impulses through real‐time feedback or stress detection and offering personalized insights or exercises that could be integrated into therapy. Another promising application mentioned in both groups was using AI to detect “predictors” of treatment outcomes or symptom trajectories based on large volumes of patient data. Participants reflected on how AI could identify personal risk patterns, such as triggers of depressive episodes, by synthesizing data from sources like mood diaries, potentially informing personalized care or timely adjustments. Lastly, in Group B, AI was mentioned as a promising tool for “monitoring” data during treatment, such as blood glucose levels or physiological stress markers, as complementary information to patient self‐report, potentially enhancing accuracy and clinical decision‐making.

In addition to this broader subtheme, we identified three separate codes, the first being “AI more accurate/objective than human.” More specifically, participants highlighted how AI might support clinicians in recognizing their own cognitive biases and making more data‐driven choices, especially in long‐term treatment trajectories. One participant reflected on the potential of AI to increase access to care, particularly by supporting less severe cases and freeing up clinicians for more complex ones, for which we created a separate code “Help more patients.” Another opportunity noted by one participant was the societal “urgency” for (research into) AI interventions, especially in response to harmful social media environments that affect vulnerable groups.

### 4.2 Challenges

The second overarching theme focused on the challenges of integrating AI into routine clinical practice. This theme was divided into two subthemes: “adoption in practice” and “human‐AI interaction in clinical decision‐making.” Within each subtheme, several distinct codes were identified.

#### 4.2.1 Adoption in Practice

The first topic mentioned under this theme was “competing with existing platforms.” More specifically, participants expressed concerns that social media platforms such as TikTok or Instagram may compete with clinically developed AI tools, especially among younger populations. They worried that these commercial platforms are more engaging, readily available, and often lack the ethical safeguards of clinical tools, making it challenging to motivate patients to adopt therapeutic alternatives. The lack of standardized data and concerns about model generalizability (“standardization & generalizability”) were the second commonly mentioned challenges. Participants highlighted issues related to limited sample diversity, inconsistencies in data entry across institutions, and poor interoperability between systems. They also noted that both data quantity and quality are crucial, and that current datasets often lack sufficient representativeness for broader clinical application. Third, in Group B, participants emphasized the difficulty of securing adequate “funding” for the rigorous development and evaluation of AI tools. They pointed out that although high standards for clinical validation are essential, these requirements often exceed the financial and infrastructural resources available to research teams. And fourth, participants noted delays and institutional obstacles in moving from research to practice (“implementation”). That is, developments in the AI field move very quickly, while progress in clinical and research settings is much slower. By the time something is tested in a patient group, the AI field has already progressed much further. This is partly due to the numerous medical ethical reviews and privacy/legal issues involved. In this regard, the two fields were speaking different languages. Last, one participant in Group A mentioned ease of use to be a critical requirement for adoption (“usability”).

#### 4.2.2 Human‐AI Interaction in Clinical Decision‐Making

This subtheme captures the tension and interdependence between human judgment and AI‐supported input in clinical contexts. Participants addressed several key issues, including responsibility and accountability in AI‐assisted decision‐making, the explainability of AI outputs, and potential overreliance on AI tools. Some noted that, in the absence of AI, clinicians base their decisions on explainable information (“explainability”) and take full responsibility (“responsibility/accountability”) for outcomes. Others pointed out that clinical decisions without AI are not always fully explainable either. Participants furthermore reflected on the ethical dilemma that highly accurate yet non‐explainable AI outputs may at times be preferable, given that the human mind is not always capable of grasping the complexity that AI systems can process. Lastly, Group A discussed overconfidence in AI tools, especially in young clinicians (“too much trust”).

### 4.3 Concerns

As already explained above, we made a distinction between “challenges” and “concerns,” where concerns are more fundamental worries than challenges, often with ethical implications. “Ethical & safety considerations” were therefore grouped under this subtheme, as well as “data sharing” between institutions, and legal questions and liability (“legal/liability”).

Participants described a wide range of “ethical and safety considerations” that are specific to the mental healthcare context. Both groups mentioned numerous fundamental values potentially at stake, such as autonomy, bias, discrimination, privacy, and transparency, and suggested that current practices may not be fully ready for AI integration. They emphasized that different types of AI applications, such as clinical support tools versus direct‐to‐patient interventions, may require fundamentally different ethical frameworks. “Data sharing” between institutions was described as both technically, ethically, and institutionally difficult. “Legal(/liability)” questions were raised exclusively by Group B. Participants noted that even anonymized or decentralized data use could lead to lawsuits.

### 4.4 Solutions

A subtheme that emerged organically during the discussions was “solutions.” Participants proposed these strategies in direct response to the challenges and concerns discussed earlier. Three recurring strategies were “human in the loop,” “collaboration,” and “train clinicians.”

Participants in both groups emphasized the importance of maintaining human oversight in AI‐supported clinical decision‐making. AI should be supportive rather than autonomous. Collaborations between different implementation partners, such as tech developers, clinicians, researchers, and platforms, were seen as essential for developing AI tools that are ethically sound and clinically relevant in both groups. These partnerships were not framed as a challenge, but rather as a key solution to existing barriers such as ethical concerns, technical limitations, and the lack of adoption in clinical practice (see also “Adoption in practice”). As the two fields are speaking different languages, participants emphasized that these kinds of developments can only be carried out collaboratively, ideally with clearly defined agreements on responsibilities, intellectual property, and involvement of implementation partners such as patients and universities. Lastly, educating clinicians was seen as a key condition for safe and effective AI implementation (“train clinicians”).

### 4.5 Evidence Needed/Evaluation

Participants in both groups emphasized the importance of robust evaluation processes to ensure that AI tools used in the treatment of eating disorders are both safe and effective. This theme consisted of two codes: “safe & accurate” and “testing & validation.”

Particularly in Group A, participants stressed the need for confidence in the accuracy and safety of AI tools before implementation in clinical practice. Group B echoed this, though less frequently, emphasizing, for example, the clinical risks of misinformation from a chatbot. Participants in both groups underlined that AI tools should undergo thorough scientific testing and validation before being used in practice.

## 5 Discussion

This qualitative study explored the perspectives of professionals working with AI and eating disorder professionals on the opportunities, challenges, and ethical considerations surrounding AI implementation in eating disorder care. Participants discussed these themes in two interdisciplinary focus groups. Participants described several use cases with a wide range of opportunities, particularly in enhancing efficiency, supporting treatment delivery, and improving clinical decision‐making through predictive models. At the same time, they identified practical challenges related to implementation and raised more fundamental concerns involving ethics, legal responsibility, and patient safety. Importantly, these issues were not discussed in abstract terms: participants proposed several solutions, including human oversight, interdisciplinary collaboration, and clinician training. The repeated emphasis on safety, accuracy, and explainability underscores that openness to AI adoption is closely tied to the perceived trustworthiness and transparency of AI tools.

The most frequently mentioned subtheme under “Opportunities” was the use of AI to reduce administrative burden. This was specifically reflected in the subcode “efficiency/administration.” This was perceived as a low‐risk, technically feasible application that could free up time for direct patient care. Reducing administrative burden is an important factor in reducing clinician burnout (Pavuluri et al. 2024). Administrative AI tools, such as AI‐assisted triage (Ilicki 2022), intake automation, or transcription services (Wang et al. 2025; Kernberg et al. 2024), are already being tested in several healthcare domains. While long‐term effectiveness studies are still emerging, the increasing institutional interest in these tools reflects a growing belief that administrative AI is one of the most viable entry points for safe, low‐risk experimentation with AI in clinical care. Participants viewed these as suitable entry points for clinical AI integration, consistent with the logic of early innovation cycles, where small‐scale pilots evolve into scalable solutions.

### 5.1 Practical Challenges to Implementation

Operationalizing these tools requires foundational infrastructure. Participants emphasized the need for adequate funding, not only to support the development and customization of AI tools, but also to ensure their integration into different clinical workflows. Without alignment in clinical workflows, IT systems, documentation standards, and privacy protocols, implementation remains fragmented. This discussion highlights the need for system‐level harmonization before even low‐risk AI tools can be reliably integrated into eating disorder care.

More complex applications, such as predictive models to support clinical reasoning, AI‐supported intervention delivery, monitoring, and other intervention tools, were met with greater caution. Under the theme “Challenges,” participants pointed to the lack of data standardization and limited model generalizability, especially when trained on narrowly defined patient groups. Models trained on highly specific datasets may fail to translate across different treatment settings, patient populations, or national contexts, which undermines both their scalability and clinical reliability. Illustrative of this problem, (Obermeyer et al. 2019) showed that a health risk prediction algorithm in the United States systematically underestimated the needs of black patients due to biases in the data.

Achieving standardization involves more than technical infrastructure: it requires collaboration between clinicians, data scientists, and institutional partners. We note that initiatives such as the Observational Medical Outcomes Partnership (OMOP) common data model (see Reinecke et al. 2021), as seen in somatic medicine, illustrate how cross‐site harmonization might be achieved. Such models may offer a potential roadmap for similar developments in mental health care regarding eating disorders. This could be a crucial stepping stone, particularly given the low prevalence of eating disorders compared to other (psychiatric) conditions. To ensure sufficiently diverse and representative training data, the development of cross‐institutional, even cross‐national, databases will be essential. This aligns with calls in the literature to overcome data fragmentation and privacy constraints through solutions such as synthetic data generation and inter‐institutional collaboration (Norris et al. 2024).

Participants also highlighted the mismatch in pace between innovation in the technological sector and implementation in clinical settings. AI developments progress rapidly, whereas clinical implementation is slowed by medical‐ethical reviews, privacy regulation, and institutional constraints. Participants noted that by the time something is tested in a patient group, the field has already moved on. Delays in implementation, combined with funding limitations and integration barriers, were described as major obstacles. At the same time, collaboration between technical and clinical partners was presented as a solution to narrow this gap and ensure clinically relevant innovation, particularly given the already noted language and perspective differences between the fields. Adoption was further complicated by usability concerns and the influence of consumer‐facing social media platforms (e.g., TikTok, Instagram), which were perceived as potentially competing with clinically developed apps/tools, especially among younger populations.

### 5.2 Ethical and Legal Considerations

Alongside these practical challenges, participants in the focus groups raised more fundamental concerns about the ethical and legal dimensions of AI in mental health care. These included potential threats to autonomy, risks of bias and discrimination, privacy violations, and a lack of transparency. Participants emphasized that existing infrastructures may not yet be equipped to handle these risks. Data sharing between institutions, while deemed necessary for training reliable models, was described as ethically, legally, and institutionally difficult. In one of the focus groups, participants raised legal questions, especially around liability in cases where even anonymized or decentralized data use could result in lawsuits.

In line with the discussion in the focus groups, we suggest that AI systems intended for psychiatric use must meet particularly high standards for safety, interpretability, and clinical benefit before widespread implementation can be ethically justified. While AI applications have gained traction in somatic medicine, such as in breast cancer screening (Chang et al. 2025) and prostate cancer diagnostics (Harmon et al. 2025), their role in psychiatry is far more complex. Nevertheless, also in somatic medicine similar concerns exist, and Chang et al. underscored the importance of retaining human oversight to minimize false positives and uphold accountability. In psychiatry, challenges are expected to be magnified, as AI systems in mental health must grapple with unstructured, highly contextual language, where subtle variations in tone, intent, or word choice can have significant clinical implications. This is further complicated by the lack of ground truth data in mental health datasets. Uncertainty in decision making among clinicians can often not be resolved with additional biological data, whereas this is possible in the case of somatic medicine. Therefore, AI models in mental health practice often lack validity (van Oosterzee 2024). Thus, AI, at this stage, should not be seen as a replacement for clinical judgment, but as a medical tool designed to augment decision‐making. Importantly, AI cannot be treated as a single, uniform concept. A distinction must be made between administrative tools, decision‐support systems, and tools that actively engage with patients in therapeutic contexts. Each of these carries different levels of risk and requires a tailored approach to evaluation and human oversight. Especially for low‐risk applications, such as administrative tools, overly cautious regulation may unnecessarily delay implementation, while more complex or autonomous systems demand stricter safeguards.

### 5.3 Human Oversight and Safeguards

Human oversight (“human in the loop”) was also discussed as a solution by the focus groups, particularly in relation to explainability and responsibility. While some participants warned that highly confident AI outputs could influence clinical decisions too strongly, especially when users lack the expertise to critically interpret them, others challenged the assumption that human decisions are necessarily more transparent or reliable. Some participants noted that AI might even outperform human judgment in specific situations, for example, by identifying early signs of treatment failure that would otherwise be missed. This led to a nuanced discussion about when explainability is necessary, and when other criteria, such as predictive accuracy or clinical usefulness, might take precedence. However, while clinical judgment is not always reliable, the clinician remains accountable. In contrast, AI outputs are not; AI can generate decisions that are logically or mathematically valid yet ethically unacceptable or, worse, hard to follow. This underlines the need for full transparency in how outputs are generated. Across both groups, there was consensus that AI should remain a decision‐support tool, not a replacement for clinical reasoning, and that safeguards are needed to avoid overreliance or the erosion of human responsibility. Next to human oversight, other solutions that were mentioned were educating clinicians on how to use AI tools. Participants also emphasized that tools must be rigorously tested and validated before implementation in practice, not only for technical performance, but also for safety, clinical fit, and usability.

### 5.4 AI Use Cases—Summary

To synthesize the use cases discussed, we distinguish between patient‐facing and clinician‐facing applications. Patient‐facing applications include chatbots for psychoeducation, relapse prevention, or symptom monitoring, as well as predictive tools that analyze speech, text, or behavioral data to detect early warning signs. While promising, these raise concerns around autonomy, therapeutic alliance, and safety if used without supervision. Clinician‐facing applications were generally seen as lower risk, focusing on automating documentation, intake, and other administrative processes to reduce workload and free time for patient care. Their implementation nonetheless requires careful attention to interoperability, privacy, and the risk of overreliance. Taken together, these use cases illustrate both the breadth of potential applications and the varying levels of risk they entail, underscoring the need for careful, context‐specific evaluation before implementation.

## 6 Limitations

This study has some limitations. First, no interview guide was used, which allowed for open, exploratory discussion but reduced consistency between groups. Second, the morning pitches may have influenced the topics and framing of subsequent conversations. Third, effort was put into decreasing chances for potential biases, but we mark the fact that some participants of the focus groups were also co‐authors of this paper. Fourth, clinical experts were overrepresented relative to technical professionals, which may have biased the discussions toward clinical priorities. In addition, some topics that were discussed, such as data sharing, apply to a more general level, not specific to eating disorder care, while funding challenges are even more pronounced in eating disorder research, where funding is inadequate, possibly related to the stigma surrounding eating disorders (Schmidt et al. 2016).

## 7 Future Research Directions

Future research should prioritize robust, context‐sensitive development of predictive AI tools for eating disorder care. A first step is to establish inter‐institutional agreements that align documentation standards and clinical indicators, enabling high‐quality data integration. Such datasets should explicitly include underrepresented patient groups to improve model generalizability and reduce bias, while transparency about data limitations and potential risks must be systematically reported. Before models are developed, a comprehensive needs assessment with all partners, including patients and families, is required. Predictive models must be built through multidisciplinary collaboration, with transparency about data limitations and the potential for bias. To ensure scientific quality and promote shared decision‐making, models should be validated across centers, made openly available where possible, and presented in ways that match patient understanding and preferences. Privacy safeguards and clarity on data ownership, liability, and intellectual property are preconditions for responsible use. Given the sensitivity of eating disorder care, predictive models should support, not replace, clinical judgment, and their use must be continually monitored for both benefits and unintended risks. Additionally, future research should prioritize validating AI tools that reduce administrative burden. As discussed, these low‐risk, technically feasible applications may increase time for direct patient care and help prevent clinician burnout.

Ensuring that AI tools are implemented responsibly in clinical settings requires more than just technical development. It calls for meaningful, ongoing collaboration between technical partners and clinicians to ensure that tools align with clinical realities, address relevant needs, and can be embedded effectively into day‐to‐day practice. To enable such collaboration, structural support is needed, including time, resources, and shared understanding across disciplines. As highlighted during the focus groups, bridging the communication gap between technical developers and clinical experts is key to ensuring AI solutions that are not only innovative but also usable and safe in practice.

Finally, several unanswered questions remain open for future investigation, including how to reconcile the rapid pace of AI innovation with slower clinical evaluation cycles, whether synthetic data can adequately address privacy restrictions without compromising validity, and how patient and family perspectives can be systematically integrated into the design and evaluation of AI systems. Addressing these issues will be critical to ensuring that AI in eating disorder care evolves in ways that are not only innovative but also safe, ethical, and clinically meaningful.

## Author Contributions

**J. Maas:** conceptualization, methodology, data curation, investigation, formal analysis, funding acquisition, writing – original draft, writing – review and editing, project administration. **S. Franssen:** methodology, formal analysis, writing – original draft, writing – review and editing. **M. Petkovic:** conceptualization, funding acquisition, writing – review and editing. **S. Cardona Cano:** writing – original draft, writing – review and editing. **A. E. Dingemans:** writing – original draft, writing – review and editing. **A. M. van Oosterzee:** writing – original draft, writing – review and editing. **M. C. T. Slof‐Op ’t Landt:** writing – original draft, writing – review and editing. **E. Talavera Martinez:** writing – original draft, writing – review and editing. **C. M. J. M. Vreeswijk:** writing – original draft, writing – review and editing. **M. Simeunovic‐Ostojic:** conceptualization, funding acquisition, writing – review and editing.

## Conflicts of Interest

The authors declare no conflicts of interest.

## Data Availability

The data that support the findings of this study are available from the corresponding author upon reasonable request.
